# Animal Models of Alzheimer’s Disease Evaluated with [^11^C]Pittsburg Compound B

**DOI:** 10.3390/life16010123

**Published:** 2026-01-14

**Authors:** Santiago Burgos-Puentes, Arturo Avendaño-Estrada, Marquiza Sablón-Carrazana, Eleazar Ramírez-Hernández, Andrea Granados-Juárez, Gerardo Bernabé Ramírez-Rodríguez, Marco Meraz-Ríos, Hilda Martínez-Coria, Miguel A. Ávila-Rodríguez

**Affiliations:** 1 Unidad Radiofarmacia-Ciclotrón, División de Investigación, Facultad de Medicina, Universidad Nacional Autónoma de México, Ciudad de México 04510, Mexico; sburgosp@uqvirtual.edu.co (S.B.-P.); arturoae@ciencias.unam.mx (A.A.-E.); 2 Centro de Investigación Sobre el Envejecimiento, CINVESTAV Sede Sur, Ciudad de México 07360, Mexico; 3 Centro de Neurociencias de Cuba, La Habana 11600, Cuba; 4 Departamento de Bioquímica, Facultad de Medicina, Universidad Nacional Autónoma de México, Ciudad de México 04510, Mexico; 5 Laboratorio de Neurogénesis, Subdirección de Investigaciones Clínicas, Instituto Nacional de Psiquiatría Ramón de la Fuente Muñiz, Ciudad de México 14370, Mexicogbernabe@inprf.gob.mx (G.B.R.-R.); 6 Departamento de Biomedicina, Centro de Investigación y Estudios Avanzados (CINVESTAV-Zacatenco), Ciudad de México 07360, Mexico; 7 Laboratorio de Neurobiología del Envejecimiento, Facultad de Medicina, Universidad Nacional Autónoma de México, Ciudad de México 04510, Mexico; hildamcoria@gmail.com

**Keywords:** beta-amyloid imaging, ^11^C-PIB, Alzheimer’s disease, AD animal models

## Abstract

Several animal models of Alzheimer’s disease have been developed and tested for diagnostic and treatment purposes. [^11^C]PIB is the gold-standard radiotracer for the detection of Aβ plaque deposits, a hallmark of the disease. This study aimed to evaluate the in vivo detection of Aβ plaques using [^11^C]PIB microPET imaging across different animal models of Alzheimer’s disease. The study included 3xTg-AD transgenic mice, TgF344-AD transgenic rats and Aβ injection-based rat model. The results showed an age-related increase in [^11^C]PIB uptake in 3xTg-AD mice, particularly in the midbrain and thalamus. In TgF344-AD rats, differences were also observed compared to WT controls, with the highest values observed in the hippocampus and cortex. In the injection-based model, inoculated rats showed greater uptake in the injection site than SHAM animals. Across all microPET studies, [^11^C]PIB uptake was consistently higher in females than in their male counterparts. These findings support the value of transgenic and Aβ injection-based models in preclinical research on Aβ plaque deposition and highlight the importance of considering species, model type, sex, and age in experimental design.

## 1. Introduction

Alzheimer’s disease (AD) is a neurodegenerative disorder that progressively and consistently leads to cognitive and memory decline, disorientation, language impairments, and behavioral changes. The pathophysiological process associated with AD begins with the accumulation of senile plaques, primarily composed of β-amyloid (Aβ) peptide deposits [1]. Positron Emission Tomography (PET) is one of the most effective imaging techniques for diagnosing and monitoring AD, and [^11^C]Pittsburgh compound B ([^11^C]PIB) is widely considered the gold standard radiotracer for the in vivo detection of βA plaque accumulation [2].

Preclinical studies in animal models are essential for the development of new radiopharmaceuticals and for evaluating their diagnostic or therapeutic effects. [^11^C]PIB has been extensively used and validated in various animal species as well as in humans [3]. However, βA deposits and their associated uptake patterns show considerable variability across different species, and even within the same species, depending on the model, sex, and age [4,5].

Several rodent models have been developed to simulate the neuropathological processes associated with Aβ accumulation. Genetically modified animals can reproduce certain features associated with the disease. Previous studies have delved into [^11^C]PIB retention in different AD mouse models, observing consistent and significant differences [4,6]. One widely used model that exhibits Aβ pathology is the triple transgenic model of AD (3xTg-AD) [7]. This model harbors three mutations: amyloid precursor protein (APPSwe), presenilin-1 (PS1M146V), and tauP301L. The 3xTg-AD mice progressively develop both Aβ pathology and tau deposition. The former begins in the cortex and progressively spreads to the hippocampus with aging [8]. PET imaging with [^11^C]PIB in 3xTg-AD mice has been used in previous studies with contradictory results. While some reported increased brain uptake in transgenic mice compared to WT controls [9], others found no significant differences in cerebral [^11^C]PIB uptake between transgenic and WT male mice [10].

While mouse models are useful and easily manipulated, their small brain size has led other studies to favor rat models, as they facilitate surgical procedures and imaging analysis. Both genetic and non-genetic rat models of AD have been developed [11]. One such model is the TgF344-AD rat model [transgenic (Tg), Fisher 344 (F344)], which expresses mutant human APPSwe and PS1 carrying the exon-9-deleted variant (PS1ΔE9). This model exhibits age-dependent cerebral amyloidosis, primarily in the hippocampus and cortex [12]. Previous studies conducted in TgF344-AD rats using [^11^C]PIB have demonstrated higher brain uptake compared with WT rats, specifically in the cortex, cerebellum, and hippocampus [13]. Although transgenic models most closely resemble human Aβ pathology, biological variability can make plaque detection unreliable in many cases. In contrast, chemical-induced AD rat models ensure the formation of Aβ deposits. Injection of the Aβ peptide replicates the pathological hallmarks of the disease and provides a specific model for screening Aβ radiopharmaceuticals [14].

Aβ-PET data can be determined using either semi-quantitative or quantitative methods, depending on the acquisition protocol and the desired quantification accuracy. A ratio-based approach demonstrated that using a simple ratio with a reference region characterized by non-specific uptake correlates well with quantitative approaches such as simplified compartmental models, providing reliable values without the need for long acquisition times [15].

This study aimed to evaluate the in vivo detection of Aβ plaques using [^11^C]PIB PET imaging across different animal models of AD. A semiquantitative analysis of microPET images was performed using the ratio method to assess Aβ plaque accumulation. Immunofluorescence studies were also performed to confirm the presence of Aβ plaques and to compare them with the microPET images qualitatively. The results were compared across species, age and sex to determine whether the models included in this study are suitable for in vivo detection of βA plaque deposition.

## 2. Materials and Methods

### 2.1. Radiotracer Synthesis

[^11^C]PIB was synthesized using the “wet” method at the Unidad Radiofarmacia-Ciclotrón, Facultad de Medicina, UNAM, in a Trasis All-in-One synthesizer (Trasis, Ans, Belgium) using a single-use cassette and a commercially available reagent kit [16]. Briefly, carbon-11 was produced in an 11 MeV cyclotron (Siemens/CTI, Knoxville, TN, USA) in the chemical form of CO_2_, reduced to [^11^C]methanolate with the aid of LiAlH_4_, and converted to [^11^C]methyl iodide ([^11^C]CH_3_I) by adding hydrochloric acid. [^11^C]CH_3_I was passed through a silver triflate reactor at 190 °C to make the conversion to [^11^C]methyl triflate ([^11^C]CH_3_OTf), then flushed through a Sep-Pak tC18 cartridge (Waters Corporation, Milford, MA, USA) pre-loaded with 1 mg of the chemical precursor 6-OH-BTA-0 (ABX Advanced Compounds, Radeberg, Germany), obtaining [^11^C]PIB in radiochemical yield of 12.7 ± 0.9% n.d.c. (n = 5) with radiochemical purity >98%.

### 2.2. Animals

For comparison, three groups were considered: (i) 3xTg-AD transgenic and wild-type (WT) control mice, (ii) TgF344-AD transgenic and WT control rats, and (iii) Aβ injection-based and SHAM control rats. The first group consisted of seven 3xTg-AD mice (5 males and 2 females), aged 8–23 months (weight: 32.9 ± 2.5 g). Additionally, two WT male mice aged 8 and 23 months were included as controls in this group. The second group consisted of eight (4 males and 4 females) double-transgenic F344 rats expressing mutant human APP and PS1 (TgF344-AD) aged 21 months (weight: 304.4 ± 65.4 g). Genotyping confirmed the presence of both transgenes in all transgenic animals. Three WT-F344 rats (2 males and 1 female) of the same age were used as controls. The transgenic mice were obtained from the animal facility of the Facultad de Medicina at Universidad Nacional Autónoma de México (UNAM), while the transgenic rats were from the animal facility of the Centro de Investigación y de Estudios Avanzados (CINVESTAV).

The Aβ injection-based model (aggregated Aβ_1-42_—Stereotaxic surgery) group consisted of six adults Wistar rats (3 males, 3 females, weight: 342.9 ± 61.6 g) obtained from the animal care facilities of the Facultad de Medicina at UNAM. Aβ_1-42_ solution was aggregated and injected into animals following the procedures and guidelines described in [17,18]. Briefly, each animal was bilaterally injected with 2 μL of Aβ_1-42_ solution, infused for 5 min using an infusion pump (Nanomite, Harvard Apparatus, Holliston, MA, USA) and a Hamilton microsyringe (0.2 μL/min). Coordinates employed were AP: −4.0 mm from Bregma; L: ±2.8 mm from midline; and DV: −2.3 mm below dura [19]. Five minutes were allowed for solution diffusion. Proper post-operative care was provided until the animal fully recovered. This group also included six SHAM rats (3 males, 3 females, weight: 318.2 ± 28.6 g) that underwent the same injury (cannula insertion) but did not receive the peptide injection. Animals were housed in groups of 3 to 5 in acrylic cages with ad libitum access to water and food, under controlled temperature (22 ± 2 °C) conditions, in 12-h light/dark cycles (light on at 8:00 a.m.). The characteristics of each group are summarized in Table 1.

All procedures described in this study complied with the technical guidelines for the production, care, and use of laboratory animals as established by SAGARPA México (NOM-062-ZOO-1999), the Institutional Committee for Care and Use of Laboratory Animals (CICUAL), and the National Institute of Health’s Guide for the Care and Use of Laboratory Animals. All efforts were made to minimize animal suffering and reduce the number of animals used. This animal study protocol was approved by the Research Ethics Committees, Research Division of the Faculty of Medicine, UNAM, Mexico (project number 65/11 August 2022).

### 2.3. Immunofluorescence Staining

Tissue processing was carried out according to the requirements of each experimental model. Brains from TgF344-AD rats were dissected, rinsed briefly in cold phosphate-buffered saline (PBS, pH 7.4), and fixed in 4% paraformaldehyde for 48 h. Then, they were cryoprotected in 30% sucrose until they sank and coronally sectioned at 30 μm using a sliding microtome (Leica Microsystems, Wetzlar, Germany). The free-floating sections were stored at 4 °C in a cryoprotective solution containing 25% ethylene glycol and 25% glycerol in 0.05 M phosphate buffer. Brains from the 3xTg-AD and pharmacologically induced models were fixed in formaldehyde for 24 h, dehydrated through an ethanol series, embedded in paraffin, and sectioned at 10 μm using a rotary microtome.

Free-floating sections from TgF344-AD rats and paraffin-embedded sections from the 3xTg-AD and pharmacologically induced models were processed following a unified protocol. Paraffin sections were first deparaffinized and rehydrated, whereas free-floating sections were processed directly to antigen retrieval. In all cases, antigen retrieval was performed by incubating the tissue in citrate buffer (pH 6.0) at 90 °C for 30 min, followed by two washes in cold citrate buffer and three washes in Tris-buffered saline (TBS, pH 7.4), each for 10 min. Sections were then incubated for 30 min in a blocking solution containing 3% normal serum and 1% Triton X-100 in TBS (Merck KGaA, Darmstadt, Germany). All sections were incubated overnight at 4 °C with mouse anti-βA primary antibody (4G8, 1:3000; BioLegend, San Diego, CA, USA). After rinsing, sections were incubated for 4 h at room temperature with Alexa Fluor 647 conjugated anti-mouse secondary antibody (1:250; Jackson ImmunoResearch, Baltimore, PA, USA). Nuclei were counterstained with DAPI, and slides were mounted using antifade mounting medium (Aqua mount, BioTnA, Kaohsiung, Taiwan).

Immunofluorescence images were acquired using a ZEISS LSM 900 confocal microscope (Carl Zeiss Microscopy GmbH, Jena, Germany). For each animal, a representative whole coronal brain section was imaged at 10× magnification using tile scanning. Z-stacks consisting of 7 optical sections with a 1 μm step size per tile were obtained. Orthogonal projections were generated to enhance spatial resolution and visualization of β-amyloid plaque distribution. Images were acquired using 405 nm and 640 nm laser lines at 3–5% power, with gain and offset adjusted during pilot sessions and subsequently fixed for all acquisitions. The pinhole was set to 1 Airy unit, and the z-step interval of 1 μm was kept constant across all samples.

### 2.4. MicroPET Imaging

Imaging was performed using a Focus 120 microPET scanner (Siemens/CTI, Knoxville, TN, USA). [^11^C]PIB was administered intravenously via the tail vein as a slow bolus injection. Doses administered were 16.9 ± 7.0 MBq for 3xTg-AD mice, 32.5 ± 11.2 MBq for TgF344-AD rats, and 36.7 ± 2.9 MBq for chemically induced rats, all following the same procedure. MicroPET scans for the latter group were performed one month after the peptide injection. All animals were anesthetized with 5% isoflurane in 100% O_2_ and maintained with 2–3% isoflurane throughout the scan, and physiological parameters (respiration, heart rate and temperature) were monitored during the scan acquisition with a Physiological Monitoring System (Harvard Apparatus). Brain PET data were acquired 30 min post-injection over a 10-min static scan and reconstructed with a two-dimensional ordered subset expectation maximization (2D-OSEM) algorithm on a matrix of 128 × 128 pixels, including corrections for scanner normalization, detector dead time, as well as random and scatter events. Additionally, dynamic PET acquisitions were performed in six mice (WT: n = 2; 3xTg-AD: n = 4) for 60 min following the injection of [^11^C]PIB, using the following frame sequence: 1 × 10 s, 4 × 30 s, 3 × 60 s, 5 × 180 s, and 4 × 600 s.

### 2.5. PET Image Analysis

Processing and analysis of the reconstructed images were performed using PMOD software (v4.3, PMOD Technologies LLC, Zürich, Switzerland). Mouse data were spatially normalized and co-registered to the Ma-Benveniste-Mirrione CT-based [Computed Tomography] anatomical atlas [20] available in the software. Uptake ratios were calculated using a reference region of 2.16 mm^3^ centered in the cerebellum, as this region is considered devoid of Aβ plaques and has been used as a reference in previous studies [21,22]. The uptake in each target region was divided by the uptake in this reference region, and one was subtracted [R = (Region_Interest_/Region_reference_) − 1]. Ratio values were computed for the midbrain (11.768 mm^3^) and thalamus (28.2 mm^3^).

Quantitative analysis was carried out for the acquired dynamic data in mice, using the Logan Reference Tissue Model (LRTM) implemented also in PMOD software. In this case a spherical volume (r = 0.8 mm) placed in the cerebellum was used as the reference tissue to obtain binding potential (BP) values to be compared with the R-values.

Rat data were spatially normalized and co-registered to an PMOD atlas based on Sprague-Dawley rat brains (A. Schwarz) MRI-based [Magnetic Resonance Imaging] anatomical atlas [23]. The reference region consisted of a centrally located cerebellar volume (14.137 mm^3^). Uptake ratios for transgenic rats were calculated for the hippocampus, parietal cortex and thalamus; for the Aβ injection-based model, the regions included the hippocampus, thalamus, and the precise site of the injury.

Error bars shown in figures represent the standard deviation derived from the regional quantification analysis performed with PMOD and reflect the uncertainty within each individual regional value rather than inter-subject variability.

## 3. Results

**Figure 1:**
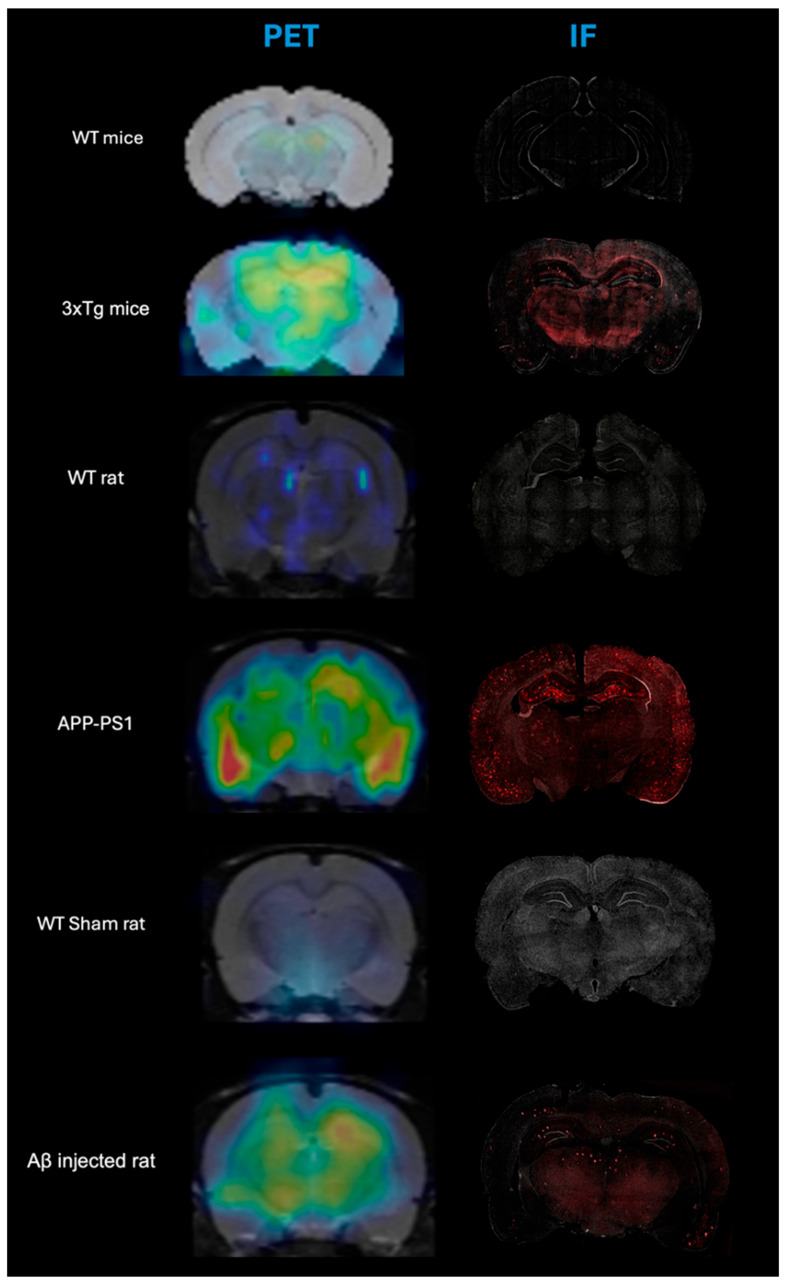
Comparative [^11^C]PIB PET/MRI and immunofluorescence (IF) images of coronal slices of the evaluated animal models and their controls. For microPET images, warm colors mean high uptake of [^11^C]PIB while in the IF, the red dots are the accumulation of amyloid plaque detected in the tissue.

Illustrative comparison between microPET and Immunofluorescence images for the evaluated animal models and their controls are shown in Figure 1, which confirms the presence of βA plaque deposition in the evaluated animal models, and its absence in the brain of control animals.

### 3.1. 3xTg-AD Mice

**Figure 2:**
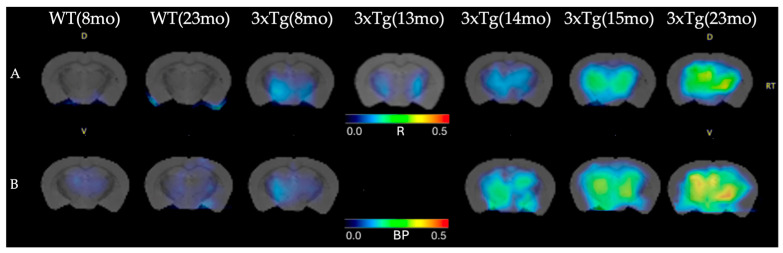
MicroPET images in (**A**) (R = ratio − 1)-scale and (**B**) BP scale of WT and 3xTg-AD male mice aged 8–23 months (mo). WT controls mice show no [^11^C]PIB uptake, whereas transgenic 3xTg-AD mice exhibit radiotracer accumulation that increases with age. The uptake pattern is comparable across both scales. The 3xTg (13 mo) mouse did not undergo a dynamic PET acquisition.

The images obtained after ratio scaling operation for male transgenic mice are shown in Figure 2A. A noticeable increase in [^11^C]PIB uptake with age can be observed. Although the ratio-based method has been used in previous studies and shown to be minimally biased and highly correlated with quantitative parameters [15,24], in addition to the ratio method analysis, kinetic modeling was applied to the dynamic data acquired in mice to perform quantitative analysis for comparison. The obtained BP parametric maps using the LRTM also showed a clear age-dependent increase in [^11^C]PIB uptake (Figure 2B), which supports the ratio-based approach used in this study.

**Figure 3:**
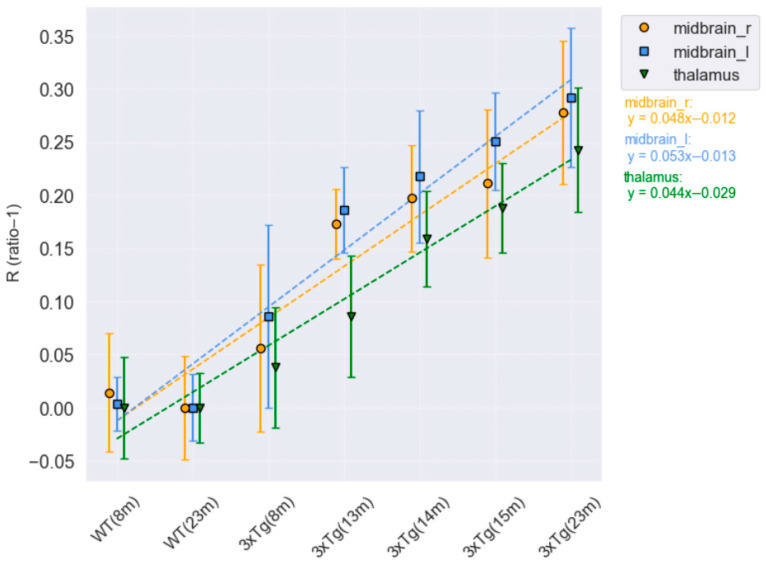
(R = ratio − 1) calculated for WT and 3xTg-AD male mice in the right and left midbrain and thalamus. WT mice show very low values compared to transgenic mice, whose values also increase with age.

The calculated values corroborate the visual observations as shown in Figure 3. R values in the midbrain (right and left) and thalamus increased with age in 3xTg-AD mice, reaching their highest values in the oldest mouse evaluated (23 months): right midbrain (0.278), left midbrain (0.292), and thalamus (0.243).

A strong correlation was found between R-values obtained by ratio-based method and BP obtained by LRTM (Pearson r = 0.975, *p* = 0.001; Spearman ρ = 0.943, *p* = 0.005). Bland–Altman analysis revealed a small bias (+0.158), indicating high consistency between the two quantification approaches, which validate the ratio-based method.

**Figure 4:**
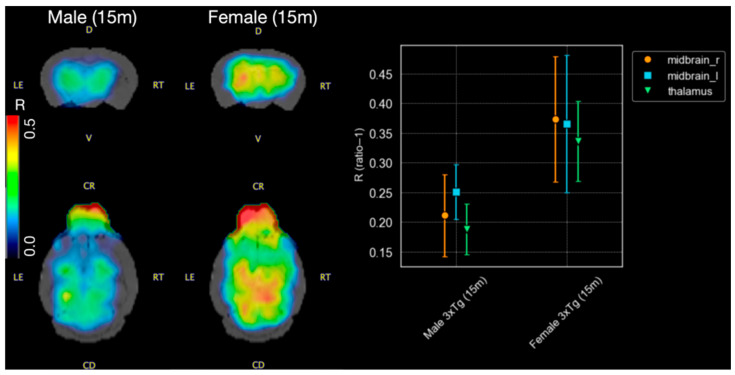
MicroPET images in (R = ratio − 1)-scale and corresponding values for a 15-month-old 3xTg-AD male mouse and female of the same age. The results show a higher Aβ burden in the female compared to the male counterpart.

When comparing a 15-month-old male and female 3xTg-AD mouse, both the scaled images and R values reveal a sex-related difference in activity concentration (Figure 4). [^11^C]PIB uptake is higher in the female mouse (right midbrain: 0.373, left midbrain: 0.365, thalamus: 0.336) compared to the male (right midbrain: 0.211, left midbrain: 0.250, thalamus: 0.188). Although the sample size is small for a formal statistical analysis, exploratory evaluation suggests that the ratio values in female 3xTg-AD mice tend to be higher (midbrain: 0.257 ± 0.132, thalamus: 0.213 ± 0.175) than those in male 3xTg-AD mice (midbrain: 0.195 ± 0.076, thalamus: 0.143 ± 0.081) across all evaluated regions.

### 3.2. TgF344-AD Rats

**Figure 5:**
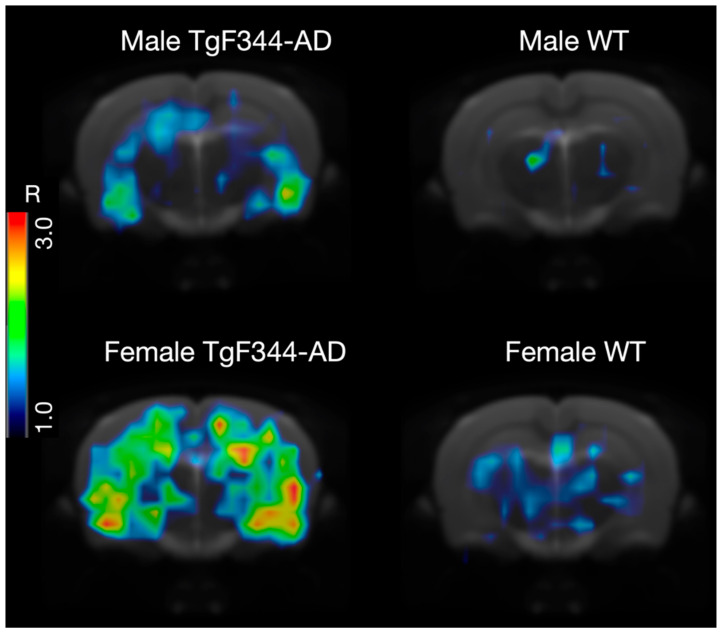
MicroPET images in (R = ratio – 1) scale and corresponding values for TgF344-AD and WT control female rats (**bottom** row), and TgF344-AD and WT and male rats (**top** row), all at 21 months of age. Notably higher [^11^C]PIB uptake is observed in transgenic animals compared to WT control animals. Moreover, the TgF344-AD female rat shows greater uptake than its male counterpart.

Results for female rats show clear differences between WT and TgF344-AD rats. [^11^C]PIB uptake is higher in transgenic rats compared to the WT control rats, as illustrated in Figure 5.

**Figure 6:**
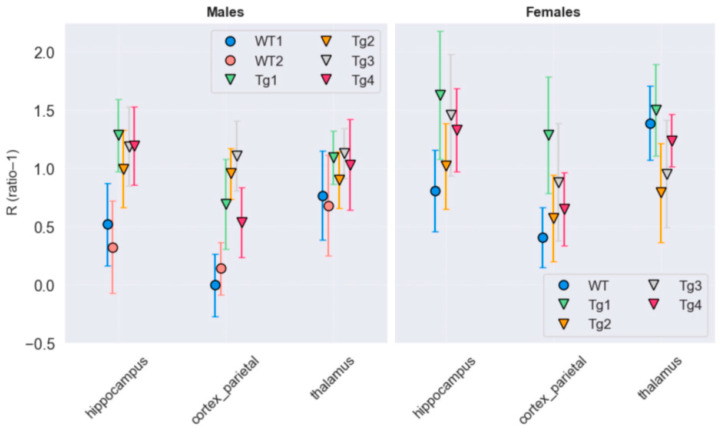
Values for TgF344-AD and WT male rats (**left** column), and TgF344-AD and WT and female rats (**right** column), all at 21 months of age. The hippocampus shows the highest values in Tg rats compared with the WT controls, while no major differences are seen in the parietal cortex and thalamus across groups. The values displayed for each VOI were obtained by averaging the values from both hemispheres. Each data point corresponds to an individual animal.

As illustrated in Figure 6, the highest R values were observed in the hippocampus of Tg female rats (left hippocampus = 1.31 ± 0.28, right hippocampus = 1.41 ± 0.25), representing a marked increase relative to the values obtained for the WT female rat (left hippocampus = 0.82, right hippocampus = 0.81). Values in the parietal cortex also showed differences between WT and Tg female rats (left parietal cortex: 0.89 ± 0.33, right parietal cortex: 0.81 ± 0.35 in Tg; left parietal cortex: 0.40, right parietal cortex: 0.42 in WT). No notable differences were found in thalamic values between WT and Tg female rats (left thalamus: 1.13 ± 0.36, right thalamus: 1.12 ± 0.28 in Tg; left thalamus: 1.40, right thalamus: 1.38 in WT).

R values in the hippocampus of male rats are also higher in transgenic animals (left hippocampus: 1.26 ± 0.16, right hippocampus: 1.07 ± 0.09) than those calculated for WT male rats (left hippocampus: 0.48 ± 0.12, right hippocampus: 0.37 ± 0.15). R values in the thalamus of male rats are high in both groups (left thalamus: 1.08 ± 0.09, right thalamus: 1.01 ± 0.13 in Tg; left thalamus: 0.70 ± 0.01, right thalamus: 0.75 ± 0.13 in WT), with no substantial differences between them.

Values in TgF344-AD female rats tend to be higher in all evaluated regions (hippocampus: 1.36 ± 0.26, parietal cortex: 0.85 ± 0.32, thalamus: 1.12 ± 0.31) compared to transgenic male rats (hippocampus: 1.17 ± 0.12, parietal cortex: 0.82 ± 0.26, thalamus: 1.04 ± 0.10). No significant differences were found between groups using a *t*-test (*p* > 0.05).

### 3.3. Aβ Injection-Based Model in Rats

**Figure 7:**
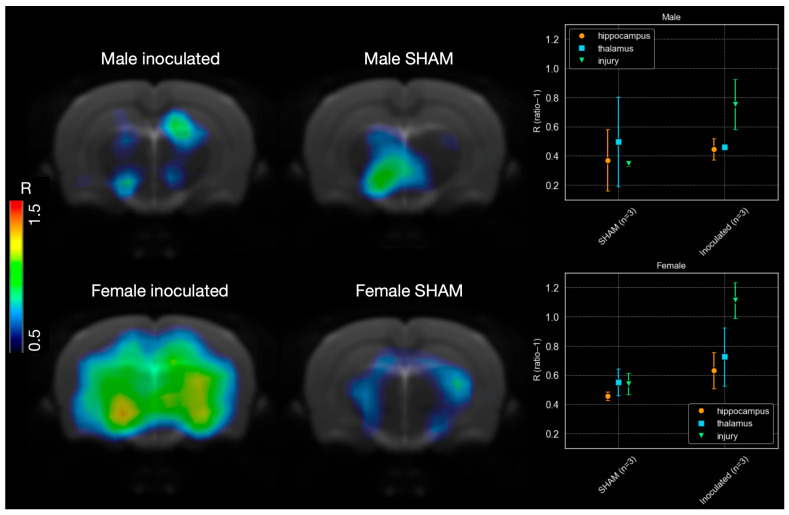
MicroPET images in R (ratio − 1) scale and corresponding values for SHAM (n = 3 males and n = 3 females) and inoculated (n = 3 males and n = 3 females) rats. Differences in [^11^C]PIB uptake are observed; the inoculated rats exhibit high radiotracer uptake in the injury site, hippocampus, and thalamus, whereas the SHAM rats show no appreciable uptake in these brain regions. The inoculated female rat exhibits higher uptake than her male counterpart. Error bars represent the standard deviation of the values for each region.

The scaled microPET images and the calculated R values revealed differences between rats inoculated with Aβ aggregates and the SHAMs (Figure 7). High values were calculated in the injury volume (R = 0.75 ± 0.17 for male, R = 1.11 ± 0.12 for female). In addition to the injury site radiotracer activity was also detected in the hippocampus (R = 0.45 ± 0.07 for male, R = 0.63 ± 0.12 for female) and thalamus (R = 0.46 ± 0.01 for male, R = 0.95 ± 0.20 for female). R values across all evaluated regions were lower for the SHAM female animals (injury site: 0.54 ± 0.07, hippocampus: 0.46 ± 0.03, thalamus: 0.55 ± 0.09). In the male rats, differences between the inoculated and SHAM animals were observed only at the injury site (injury site: 0.34 ± 0.02, hippocampus: 0.37 ± 0.21, thalamus: 0.50 ± 0.31). The inoculated female rats exhibited higher R-values in all evaluated regions than the male rats (Mann–Whitney U test, *p* = 0.007).

## 4. Discussion

In this study, a semiquantitative analysis of [^11^C]PIB cerebral uptake was performed across different animal models of AD to evaluate Aβ plaque accumulation using the ratio method, with a volume centered in the cerebellum as the reference region. Although interspecies differences and nonspecific binding cannot be completely excluded, the cerebellar volume appears to provide a consistent reference tissue for ratio-based quantification, for the models evaluated in this work. 3xTg-AD mice showed a progressive increase of [^11^C]PIB uptake with age, consistent with the gradual accumulation of Aβ deposits characteristic of AD pathology in this animal model. The result aligns with previous reports indicating that, in 3xTg-AD mice, extracellular Aβ deposits begin to appear around six months of age and become more prominent by 12 months or later [25]. However, the R values calculated did not indicate an increase in the cortex, as would have been expected based on the known neuropathology of this transgenic model. Instead, higher values were observed in other regions, such as the midbrain and thalamus. A previous study did not detect significant differences in amyloid deposition using microPET imaging with the same radiopharmaceutical [10]. In contrast, our results reveal prominent uptake in the midbrain and thalamus, becoming clearly visible from 14 months of age.

Previous studies have reported that Aβ burden increases significantly with age in transgenic female mice and tends to be significantly greater than that observed in male counterparts [26,27]. In this study, both male and female 3xTg-AD mice at 15 months of age were evaluated, and a higher [^11^C]PIB uptake was observed in the transgenic female mouse. These findings support prior evidence and underscore the importance of accounting for sex differences in Aβ-PET imaging studies.

The transgenic rat model (TgF344-AD) also showed differences compared to WT control rats. The highest [^11^C]PIB uptake was observed in the hippocampus, cortex and thalamus. The thalamus should not be considered a distinguishing region between transgenic and WT animals, as no important differences were observed in this area. Previous studies have reported similar findings in TgF344-AD rats using [^11^C]PIB, showing continuous deposition from 6 months of age in the hippocampus and temporoparietal cortex [28]. The thalamic activity found in this work in both 3xTg-AD and control mice, and rats, has not been previously reported. However, it is important to note that both the 3xTg-AD and TgF344-AD animal models express the human APP and PSEN1 mutations [8,12]. In mice carrying PS1- APP mutations, large Aβ plaques have been reported to develop in the thalamus from around 8 months of age [29]. This could be related to the high signal detected in the thalamus of these animal models; however, other mechanisms might also be involved. For instance, it is known that [^11^C]PIB not only reflects Aβ load but also tracer delivery. Therefore, the uptake observed in regions not typically associated with amyloid pathology may also be related to changes in the blood–brain barrier or regional perfusion differences.

In humans, [^11^C]PIB uptake has been observed in the thalamus in both AD-positive subjects and healthy controls. Only in the advanced stages of the disease, significant differences between groups become apparent [30]. A similar phenomenon could occur in these animal models, where part of the [^11^C]PIB uptake might result from non-specific binding to proteins or lipid structures located within thalamic regions, which could also explain the signal detected in control animals.

Compared with values in their female counterparts, all values in transgenic male animals are slightly lower, further supporting the consistently higher Aβ deposition observed in females. The mechanisms underlying the differences between males and females have not been fully elucidated. However, recent studies have shown that hormonal levels influence amyloid burden. Specifically, estrogens exert a neuroprotective role by reducing Aβ production and increasing its degradation. During aging, females experience a progressive reduction in the levels of sexual hormones, which could partially explain the higher Aβ plaque burden observed in females [31,32].

In the Aβ injection-based model, [^11^C]PIB uptake was evident at the injection site in both male and female inoculated rats, supporting the tracer’s specificity for Aβ deposits. In female inoculated rats, radiotracer activity was also observed in regions such as the hippocampus and thalamus. These results support the hypothesis that intracerebral injection of Aβ_1-42_ peptide can accelerate in vivo Aβ plaque formation [33]. The values obtained for the SHAM female animal were lower across all evaluated regions compared to the inoculated female rat. In the male rat, differences between the inoculated and SHAM animals were observed only at the injury site. As previously observed across other animal models, R values in inoculated male rats were lower than those in females.

Table 2 provides a summary of the key brain regions evaluated in each model, including the AD-model with their corresponding controls and their R_mean_ values. The R_mean_ values were obtained by averaging the values within each group without separating by sex.

Among the models included in this study (3xTg-AD mice, TgF344-AD rats, and Aβ-injected rats), the highest R values and the most distinct [^11^C]PIB uptake patterns were observed in female TgF344-AD rats. These findings highlight this model as a suitable option for detecting Aβ plaque pathology via microPET imaging and suggest it may serve as a robust and reliable tool for preclinical evaluation of AD-related amyloid pathology.

In all animal models of Alzheimer’s disease evaluated in this research, the IF images showed antibody labeling in regions that also showed signal in the PET images. This match confirms the presence of Aβ deposits in the animals included in this study with AD model and highlights the added value of microPET imaging as a sensitive in vivo technique for the specific detection of Aβ deposits using [^11^C]PIB.

### Limitations

The main limitation of this study is the relatively small sample size (n), which may reduce the statistical power of the findings. However, it is important to emphasize that this work was designed as a proof-of-concept, with the primary objective of comparing different animal models of Alzheimer’s disease rather than quantifying beta-amyloid accumulation.

## 5. Conclusions

All PET studies conducted here with transgenic animals support previous findings reporting sex-related differences, showing higher uptake in female animals compared to males. In 3xTg-AD mice, results suggest an age-dependent uptake of [^11^C]PIB, and was consistent with both methods evaluated in this work for the Tg mice. R values calculated in TgF344-AD rats were higher compared to WT control rats. In the Aβ injection-based rat model, brain microPET imaging showed a correlation between the [^11^C]PIB uptake and Aβ deposits, with the highest R values observed at the area of injury. These findings highlight the value of both transgenic and Aβ injection-based models for studying the in vivo progression of Aβ plaque deposition. The results obtained in this research work suggest that sex, model, and age-related differences should be taken into account in future preclinical research.

## Figures and Tables

**Table 1:** Summary of animal groups characteristics.

Tg-Mice	3xTg-AD mice	n	7 (5 males, 2 females)
		ages	Between 8 and 23 months
		weight	32.9 ± 2.5 g
	WT control mice	n	2 (males)
		ages	8 and 23 months
		weight	35.2 ± 1.6 g
Tg-Rats	TgF344-AD rats	n	8 (4 males, 4 females)
		ages	All 21 months
		weight	304.4 ± 65.4 g
	WT-F344 rats	n	3 (2 males, 1 females)
		ages	All 21 months
		weight	321.3 ± 86.3 g
Inoculated-Rats	Aβ injection-based adult rats	n	6 (3 male, 3 female)
		weight	342.9 ± 61.6 g
	SHAM adult rats	n	6 (3 male, 3 female)
		weight	318.2 ± 28.6 g

**Table 2:** Summary of main findings for the models evaluated in this work.

	Tg-Mice			Tg-Rats			Inoculated Rats		
		3xTg	WT		TgF344	WT		Aβ Rats	SHAM
		R_mean_	R_mean_		R_mean_	R_mean_		R_mean_	R_mean_
Key brain regions	right midbrain	0.200	0.014	hippocampus	1.265	0.554	hippocampus	0.537	0.413
	left midbrain	0.225	0.004	parietal cortex	0.838	0.461	injury	0.932	0.444
	thalamus	0.163	0.0	thalamus	1.082	0.948	thalamus	0.654	0.524

## Data Availability

All data generated and analyzed in the current study are available from the corresponding author on reasonable request.

## Abbreviations

The following abbreviations are used in this manuscript:

AD	Alzheimer’s disease
PET	Positron Emission Tomography
Aβ	β-amyloid
[^11^C]PIB	[^11^C]Pittsburgh compound B
3xTg-AD	Triple transgenic model of AD
APP	Amyloid precursor protein
PS1	Presenilin-1
Tg	Transgenic
F344	Fisher 344
[^11^C]CH_3_I	[^11^C]methyl iodide
[^11^C]CH_3_OTf	[^11^C]methyl triflate
WT	Wild type
UNAM	Universidad Nacional Autónoma de México
CINVESTAV	Centro de Investigación y de Estudios Avanzados
CICUAL	Committee for the Care and Use of Laboratory Animals
PBS	Phosphate-buffered saline
TBS	Tris-buffered saline
2D-OSEM	Two-dimensional ordered subset expectation maximization
CT	Computed Tomography
MRI	Magnetic Resonance Imaging
IF	Immunofluorescense
R	Ratio-1
BP	Binding Potential
LRTM	Logan Reference Tissue Model
n	Number of animals
